# Validity and reliability of an online self-report 24-h dietary recall method (Intake24): a doubly labelled water study and repeated-measures analysis

**DOI:** 10.1017/jns.2019.20

**Published:** 2019-08-30

**Authors:** Emma Foster, Clement Lee, Fumiaki Imamura, Stefanie E. Hollidge, Kate L. Westgate, Michelle C. Venables, Ivan Poliakov, Maisie K. Rowland, Timur Osadchiy, Jennifer C. Bradley, Emma L. Simpson, Ashley J. Adamson, Patrick Olivier, Nick Wareham, Nita G. Forouhi, Soren Brage

**Affiliations:** 1 Human Nutrition Research Centre, Institute of Health and Society, Newcastle University, Newcastle upon Tyne, UK; 2 School of Mathematics, Statistics and Physics, Newcastle University, Newcastle upon Tyne, UK; 3 Department of Mathematics and Statistics, Lancaster University, Lancaster, UK; 4 MRC Epidemiology Unit, University of Cambridge, Cambridge, UK; 5 MRC Elsie Widdowson Laboratory, Cambridge, UK; 6 Open Lab, School of Computing Science, Newcastle University, Newcastle upon Tyne, UK; 7 Faculty of Information Technology, Monash University, Clayton, VIC, Australia

**Keywords:** Dietary assessment, Online 24-h dietary recall, Doubly labelled water, Validation, Repeatability, Reliability, UK adults, ASA24^®^, automated self-administered 24-h dietary assessment tool, DLW, doubly labelled water, EI, energy intake, ICC, intra-class correlation coefficient, Intake24, self-completed computerised dietary recall system based on multiple-pass 24-hour recall, NMES, non-milk extrinsic sugars, RQ, respiratory quotient, TEE, total energy expenditure

## Abstract

Online self-reported 24-h dietary recall systems promise increased feasibility of dietary assessment. Comparison against interviewer-led recalls established their convergent validity; however, reliability and criterion-validity information is lacking. The validity of energy intakes (EI) reported using Intake24, an online 24-h recall system, was assessed against concurrent measurement of total energy expenditure (TEE) using doubly labelled water in ninety-eight UK adults (40–65 years). Accuracy and precision of EI were assessed using correlation and Bland–Altman analysis. Test–retest reliability of energy and nutrient intakes was assessed using data from three further UK studies where participants (11–88 years) completed Intake24 at least four times; reliability was assessed using intra-class correlations (ICC). Compared with TEE, participants under-reported EI by 25 % (95 % limits of agreement −73 % to +68 %) in the first recall, 22 % (−61 % to +41 %) for average of first two, and 25 % (−60 % to +28 %) for first three recalls. Correlations between EI and TEE were 0·31 (first), 0·47 (first two) and 0·39 (first three recalls), respectively. ICC for a single recall was 0·35 for EI and ranged from 0·31 for Fe to 0·43 for non-milk extrinsic sugars (NMES). Considering pairs of recalls (first two *v.* third and fourth recalls), ICC was 0·52 for EI and ranged from 0·37 for fat to 0·63 for NMES. EI reported with Intake24 was moderately correlated with objectively measured TEE and underestimated on average to the same extent as seen with interviewer-led 24-h recalls and estimated weight food diaries. Online 24-h recall systems may offer low-cost, low-burden alternatives for collecting dietary information.

Information on dietary intakes of individuals and populations is important in determining diet–disease associations, identifying deficiencies and excesses of nutrients, and evaluating the impact of interventions. The majority of methods for assessing the diet of individuals involve an interview with trained personnel, manual coding of foods, calculation of portion sizes, and matching to food composition tables. Therefore, such methods tend to be costly and time-consuming. With traditional methods, such as the weighed food diary, issues of compliance and under-reporting of habitual energy intake (EI)^(1,2)^, participant selection bias and recording bias^(3)^ are a significant concern.

Recent advances in technology and the ubiquity of Internet access in many countries have led to the development of web-based systems for collecting information on dietary intake remotely. These include online dietary 24-h recall systems^(4,5)^, online food diaries^(6)^ and online FFQ^(7)^. These systems can be completed at a time and place convenient to the participant, without the need for a trained interviewer, and this may reduce the respondent burden and reduce barriers to participation.

Intake24 is an online dietary recall system (https://intake24.co.uk/) which can be completed by participants remotely. Originally designed for use by people aged 11–24 years it was subsequently extended for the general adult population and tested with people aged 11–88 years^(8)^. The system is based on the multiple-pass 24-h recall^(9)^ and contains a database of over 2500 foods linked to food composition codes^(10)^. Versions are available for the UK, Portugal, Denmark, New Zealand and the United Arab Emirates, with versions for India and Australia under development. A series of food photographs are used for portion size estimation. These have previously been criterion-validated in a feeding study and also evaluated for convergent validity against weighed food diaries with children aged 18 months to 16 years and their parents^(11,12)^. Intake24 was developed through four cycles of user-testing and feedback^(13)^. Convergent validity testing of Intake24 against interviewer-led 24-h recalls found that the two methods yielded comparable estimates^(14)^; however, the instrument has not yet been criterion-validated against objective measures of energy, nor has reliability been examined.

The doubly labelled water (DLW) method is considered the reference standard to estimate free-living total energy expenditure (TEE)^(15,16)^; one of its uses has been to validate dietary EI instruments. The underlying assumption is that if participants are in energy balance, then over a period of time, total EI should be equivalent to TEE^(17)^. These comparisons have led to the observation that underestimation of food intakes is a common problem in dietary surveys^(3,18)^.

To establish the validity and reliability of the system for use in UK adults, we aimed to: (1) assess the validity of self-reported EI using Intake24 in a cohort of adults against concurrent objective measures of energy expenditure using DLW; and (2) test the reliability of estimates of energy and key nutrients using pooled data from studies for participants completing four or more recalls.

## Methods

### Validation of Intake24 reported energy intake against doubly labelled water measured energy expenditure

#### Study population and recruitment

We recruited fifty men and fifty women across three age categories (40–49 years; 50–59 years; 60–65 years) across a wide range of BMI from the Fenland Study, an ongoing population-based study in the Cambridgeshire area, UK^(19)^. A sample size of 100 participants was recruited on a first-come, first-served basis when fulfilling age/sex/BMI category eligibility and asked to attend two clinic visits. This size of sample allows estimation of the 95 % CI about ±0·34*s* (where *s* is the standard deviation of the differences between measurements by the two methods)^(20)^. Travel expenses were paid but participants did not otherwise receive any monetary incentive for taking part. Data collection for this component of the study was carried out between November 2015 and September 2016. See Supplementary Fig. S1 for the participant flow chart for the validation study. The present study was conducted according to the guidelines laid down in the Declaration of Helsinki. Ethical approval for the study was obtained from Cambridge University Human Biology Research Ethics Committee (reference no. HBREC/2015.16) and all participants provided written informed consent.

#### Doubly labelled water administration

Participants attended their first clinic visit with a (baseline) urine sample collected at least 1 d prior to their visit (sample bottles provided in their appointment letter). During this visit, a second baseline (fasting) urine sample was collected. Participants were then asked to drink a body weight-specific dose of DLW (deuterium oxide-18; D_2_^18^O) and collect daily (post-dose) urine samples for the next 9–10 d. The dose used was 174 mg/kg H_2_^18^O and 70 mg/kg^2^H_2_O. (Oxygen 18 was supplied by Sercon Ltd; deuterium was supplied by Goss Scientific Instruments Ltd, the UK distributor for Cambridge Isotope Laboratories.) The method of Schoeller was followed which fixes the space ratio to a value of 1·03^16^.

Participants were provided with labelled sampling bottles and a recording sheet and were instructed to collect one urine sample every day, at a similar time of day, at any time apart from the first void of the day. Participants were asked to record the date and time of each sample and keep the samples refrigerated until returning them at the second clinic visit following the free-living observation period. A final post-dose urine sample was obtained during the second clinic visit. All participants provided enough pre- and post-dose samples for calculation of TEE (see below). Height (cm) and weight (kg) were measured using standardised anthropometric procedures and BMI was calculated (kg/m^2^).

#### Intake24 administration

Participants were asked to complete Intake24 at least twice and ideally on three occasions during the DLW measurement period but the days on which to complete the recall were not specified. At the first clinic visit, each participant was issued with a unique username and password and provided with the URL (i.e. web address) with which they could access Intake24. If the participant had not completed at least two instances of Intake24 during the measurement period, or did not have Internet access, they were asked to complete Intake24 at the second clinic visit. Two participants did not complete Intake24 remotely. One of whom provided dietary data on paper at the second visit; these two individuals were excluded from this analysis.

#### Doubly labelled water sample analysis

Urine samples were analysed, in duplicate, for ^18^O enrichment using the CO_2_ equilibration method of Roether^(21)^. Briefly, 0·5 ml of sample was transferred into 12 ml vials (Labco Ltd), flush-filled with 5 % CO_2_ in N_2_ gas and equilibrated overnight whilst agitated on rotators (Stuart, Bibby Scientific). Headspace of the samples was then analysed using a continuous flow isotope ratio mass spectrometer (AP2003; Analytical Precision Ltd). For ^2^H enrichment, 0·4 ml of sample was flush-filled with H_2_ gas and equilibrated over 6 h in the presence of a platinum catalyst. Headspace of the samples was then analysed using a dual-inlet isotope ratio mass spectrometer (Isoprime; GV Instruments).

All samples were measured alongside secondary reference standards previously calibrated against the primary international standards Vienna-Standard Mean Ocean Water (vSMOW) and Vienna-Standard Light Antarctic Precipitate (vSLAP) (International Atomic Energy Agency). Sample enrichments were corrected for interference according to Craig^(22)^ and expressed relative to vSMOW. Analytical precisions are better than ±0·62 % for δ^18^O and ±0·5 % for δ^2^H. Please see Supplementary Calculation S1 for full details.

#### Data analysis

The method of Bland & Altman^(20)^ was used to examine accuracy (mean bias) and precision (root mean square error and 95 % limits of agreement) of reported EI by Intake24 against TEE measured using DLW. The ratio of reported daily EI (based on the first 24-h recall, the mean of the first two 24-h recalls and the mean of the first three 24-h recalls) to energy expenditure was calculated. As the data were not normally distributed, they were log-transformed. We define absolute validity by the log-ratio (log(EI/TEE)), where a negative log-ratio represents under-reporting and a positive log-ratio indicates over-reporting of EI. The ratio of the arithmetic mean is also presented along with the geometric mean to allow comparison with previous studies.

We also examined the correlation between reported EI and energy expenditure to quantify ability of the instrument to rank individuals. In addition, we examined the role of intra-individual intake variation in these correlation coefficients using data for participants who had reported at least 3 d, as described by Rimm *et al*.^(23)^. To assess whether the validity of Intake24 depended on demographic characteristics, we applied a mixed-effects model to account for multiple observations per individual, in which the dependent variable was the log-ratio, and the covariates included age, sex, height (in cm) and BMI.

### Assessment of Intake24 reliability

#### Study population and recruitment

The repeatability of measures of EI and key nutrients was examined using datasets from three previous surveys. These were a comparison of Intake24 against interviewer-led recalls in 11- to 24-year-olds (survey 1; *n* 129)^(14)^, comparison of Intake24 against interviewer-led recalls in adults aged 24–68 years (survey 2; *n* 46) and a field test of Intake24 in the Scottish Health Survey population with people aged 11–88 years old (survey 3; *n* 133)^(8)^. See Supplementary Fig. S2 for the participant flow chart for the repeatability study.

Only the initial mode of contact differed between the surveys. For survey 1, 11- to 16-year-olds were recruited from secondary schools in Dundee and Newcastle upon Tyne. The 17- to 24-year-olds were recruited by a recruitment agency who approached potential participants in the street. For survey 2, posters and leaflets were displayed in locations around Newcastle including the University campus, local shops, fitness centres and childcare facilities. Recruitment for survey 3 was conducted in collaboration with ScotCen Social Research; 1000 participants who had previously taken part in the Scottish Health Survey were sent an introductory letter and followed up by telephone.

All participants were required to give written consent (written assent and parental consent were obtained for those under the age of 18 years) before participating in the research. Ethical approval for these surveys was granted by Newcastle University's Faculty of Medical Sciences Ethics Committee (reference no. 00706/2013, survey 1; no. 01018/2016, survey 2; no. 00875/2015, survey 3).

#### Intake24 administration

For all three surveys included in the reliability analyses, participants were asked to complete Intake24 on 4 d over a 10 d period, including both week and weekend days. Participants were not aware of their scheduled days in advance but were sent an email on the day of each recall with the URL and log-in details asking them to complete a recall for the previous day's food intake.

#### Data analysis

For individuals completing four or more recalls using Intake24, we assessed both test–retest reliability of a single recall and reliability of a single-repeat recall; the latter was done by comparing the average of the first two recalls (pair 1) against the average for the following two recalls (pair 2). For both methods, intra-class correlation coefficients (ICC) and their 95 % CI were calculated using a two-way mixed-effects model for absolute agreement; this included evaluation of the influence of age and sex on reliability. Reliability is classified as poor, moderate, good or excellent based on the CI of the ICC as recommended by Koo & Li^(24)^. In addition, the method of Bland & Altman was used to assess agreement (with 95 % limits) between the first and second recall, and between the average of first two recalls and the average of following two recalls. The ratios of reported intakes were calculated for energy and key nutrients. As the data were not normally distributed they were log-transformed. The values presented are the ratios of the geometric mean. All analyses were conducted with SPSS (SPSS Statistics for Windows, version 22.0; IBM Corp.) or R (v3.4.3)^(25)^ statistical software packages. *P* values were considered statistically significant at the α = 0·05 level.

## Results

### Validation of Intake24 against doubly labelled water measures of total energy expenditure

A total of ninety-eight participants (fifty women and forty-eight men) completed at least one 24 h recall (Table 1). Demographic data for participants completing two recalls and three recalls varied only slightly. Participants ranged in age from 40 to 65 years, and had a mean BMI of 26·6 (range 20–37) kg/m^2^, with no significant change in weight over the recording period. The mean weight change for the participants was +0·09 (sd 0·80) kg, eight participants lost between 1 and 1·5 kg while twelve participants gained between 1 and 2·2 kg. The remaining seventy-five participants had a weight difference of less than 1 kg between the beginning and the end of the data collection period.

**Table 1.** Baseline characteristics of participants completing the doubly labelled water (DLW) study* (Mean values and standard deviations; minimum and maximum values)

	Mean	sd	Minimum	Maximum
Age (years)	54·3	7·30	40·0	65·0
Height (cm)	171·2	9·45	150·6	194·4
Weight (kg)	78·2	13·75	48·7	110·8
BMI (kg/m^2^)	26·6	3·47	20·4	36·6
DLW TEE (kJ/d)	12 007	2374·9	6852	17 043
EI 1 d (kJ/d)	8885	4287	1622	21 956
EI 2 d (kJ/d)	9261	4019	4175	21 979
EI 3 d (kJ/d)	9240	4008	4520	16 962
kO (/d)	0·119	0·030	0·066	0·257
kH (/d)	0·093	0·028	0·044	0·228
NO (mol)	2118	435	1215	3131
NH (mol)	2180	448	1251	3224

TEE, total energy expenditure; EI, energy intake; kO, decay constant for O; kH, decay constant for H; NO, body oxygen pool; NH, body hydrogen pool.

* The mean of the observed space ratio was 1·033 (range 1·016–1·046).

**Fig. 1.**
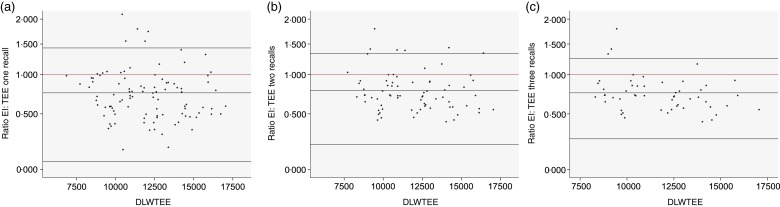
Bland–Altman plots of ratio of reported energy intake (EI) from first 24 h recall (a), mean of first two 24 h recalls (b) and mean of first three 24 h recalls (c) to total energy expenditure measured by doubly labelled water (DLWTEE).

The mean of EI from three recalls and DLW-based TEE were 9240 (sd 4008·5) and 11 670 (sd  2279·8) kJ/d, respectively, indicating under-estimation of self-reported EI by 25 % and almost twofold greater variation of self-reported EI in the population (sd  4008·5 *v.* 2279·8 kJ/d). Although reporting accuracy of the population averages did not appear to change markedly with increasing number of days recalled, the precision, as evidenced by the width of the limits of agreement (Fig. 1), improved with number of recalls.

The Bland–Altman plots (Fig. 1) show a range of under-estimation and over-estimation of EI reported using Intake24 amongst the individuals in this validation study. There is some evidence of systematic bias with an increased tendency to under-report at lower levels of intake and to over-report at higher levels of intake when EI based on a single recall is considered (Fig. 1(a)), but this pattern is no longer apparent when the mean of three recalls is used (Fig. 1(c)).

The mixed-effects model indicated no significant pattern for under- or over-reporting across BMI and sex (Table 2 and Supplementary Table S1). Age was positively associated with EI/TEE, indicating that older participants tended to under-report to a lesser extent. On average people of 40 years of age were found to under-report their EI by 42·6 % whereas in people of 60 years of age EI was under-reported by 18·7 %.

**Table 2.** Accuracy and precision of energy intakes reported using Intake24 – doubly labelled water study*

	No. of recalls	n	Energy intake (kJ/d)		TEE (kJ/d)		Correlation REI and TEE	P	Mean ratio† EI:TEE	Geometric mean	95 % Limits of agreement‡	
			Mean	sd	Mean	sd					Lower	Upper
All	1	98	8885	4287	12 006	2374	0·31	0·002	0·75	0·68	0·27	1·68
	2	74	9261	4019	11 883	2351	0·42	< 0·001	0·78	0·74	0·39	1·41
	3	53	9240	4008	11 670	2279	0·31	0·025	0·75	0·72	0·40	1·28
Men§	1	48	9799	4772	13 629	1934	0·22	0·14	0·72	0·64	0·23	1·81
	2	34	10 123	4185	13 586	1950	0·29	0·10	0·80	0·76	0·39	1·46
	3	24	10 190	4130	13 306	1877	0·19	0·37	0·73	0·70	0·41	1·21
Women	1	50	8008	3598	10 450	1588	0·25	0·082	0·77	0·72	0·34	1·52
	2	40	8434	3708	10 435	1562	0·17	0·30	0·76	0·73	0·38	1·37
	3	29	8329	3703	10 317	1601	0·07	0·71	0·77	0·73	0·39	1·36
BMI < 25 kg/m^2^§	1	35	8963	4357	10 818	2273	0·36	0·036	0·83	0·76	0·30	1·93
	2	31	9250	4131	10 916	2171	0·69	< 0·001	0·79	0·77	0·48	1·24
	3	23	9267	4144	10 657	1956	0·75	< 0·001	0·77	0·76	0·50	1·15
BMI 25–30 kg/m^2^	1	47	7942	3174	12 332	2157	0·26	0·073	0·65	0·60	0·27	1·34
	2	32	8697	3638	12 257	2236	0·23	0·21	0·80	0·74	0·35	1·57
	3	21	8717	3631	11 861	2022	−0·19	0·42	0·78	0·73	0·36	1·49
BMI > 30 kg/m^2^	1	16	11 487	5911	13 652	1992	0·27	0·31	0·84	0·74	0·26	2·12
	2	11	10 944	4602	13 520	2099	0·17	0·61	0·71	0·67	0·33	1·36
	3	9	10 721	4618	13 817	2192	0·56	0·12	0·62	0·60	0·36	1·00
40–49 years§	1	30	8217	4043	12 194	2326	0·15	0·44	0·69	0·62	0·25	1·57
	2	23	8683	4894	12 143	2271	0·27	0·21	0·67	0·65	0·36	1·15
	3	15	7499	3214	11 752	2247	0·54	0·04	0·65	0·63	0·36	1·08
50–59 years	1	34	8952	4865	11 977	2451	0·29	0·10	0·75	0·67	0·26	1·73
	2	25	9021	5395	11 767	2616	0·43	0·03	0·74	0·70	0·38	1·29
	3	19	7455	1637	12 026	2535	0·47	0·04	0·67	0·66	0·44	1·00
60–65 years	1	34	9410	3913	11 872	2402	0·50	0·002	0·79	0·74	0·32	1·71
	2	26	11 298	5346	11 764	2223	0·23	0·26	0·92	0·88	0·48	1·59
	3	19	10 395	4114	11 251	2083	0·05	0·85	0·91	0·87	0·49	1·54

TEE, total energy expenditure; REI, reported energy intake; EI, energy intake.

* Data are nested with respect to number of recalls (first recall results for everyone, first two recall results for everyone with at least two recalls, and so on).

† The ratio is the reported mean daily energy intake divided by the total energy expenditure as measured by doubly labelled water. The ratio equal to 1 would indicate exact agreement; <1, underestimation; and >1, overestimation.

‡ Derived from ±2 sd of log-transformed ratios.

§ *P* = 0·11 for the association of BMI with the ratio of reported EI to TEE; *P* = 0·91, sex difference; and *P* = 0·003, age.

Scatterplots showing EI against TEE, on both original and log scales, are provided in Supplementary Fig. S3. The correlations were 0·31, 0·42 and 0·31 for the first, first two and first three recalls (Table 2), respectively, and generally stronger in normal-weight individuals (0·69 for first two recalls) than in over-weight (0·23) and obese (0·17). The deattenuated correlation coefficients after log-transformation are 0·31 for the first recall, 0·47 for the first two recalls and 0·39 for the first three recalls, showing slight improvement after accounting for intra-individual variation.

### Assessment of Intake24 reliability

As data for the reliability analysis are pooled from several separate studies, the number of participants in each age and sex group were not balanced (Table 3).

**Table 3.** Demographics of participants included in the reliability study (data from three studies*) (Numbers of participants)

Age group	Number	Men	Women
11–16 years	87	34	53
17–24 years	124	80	44
25–64 years	68	31	37
65 years +	24	18	6
Total	303	163	140

* Test–retest reliability of energy and nutrient intakes was assessed using data from three further UK-based studies where participants aged 11 to 88 years completed Intake24 a minimum of four times, as described in the Methods section.

For most nutrients, considering the mean of a pair of recalls increased the reliability compared with a single recall administration. Pairs of two recalls produced similar population averages for energy and the macronutrients, as evidenced by mean ratios ranging from 0·99 to 1·10. Slightly poorer reliability was seen for non-milk extrinsic sugars (NMES), alcohol and vitamin C. The limits of agreement were wider for those nutrients for which intakes tend to vary more day to day. The very large limits of agreement for alcohol were due to the fact that most recall days did not include any alcohol and most participants drank on only one of the four recall days, if at all (Table 4).

**Table 4.** Reliability of reported intakes of total energy and nutrients among participants aged 11 years and over (*n* 303*)

	Single recalls								Paired recalls							
Nutrient	Mean recall 1	Mean recall 2	Mean ratio	Limits of agreement		Reliability†			Mean recall 1	Mean recall 2	Mean ratio	Limits of agreement		Reliability†		
				Lower	Upper	ICC	95 % CI					Lower	Upper	ICC	95 % CI	
Energy (kJ)	7106	6880	1·03	0·39	2·77	0·347	0·286,	0·409	7198	7000	1·03	0·53	2·00	0·516	0·428,	0·594
Fat (g)	59·6	55·9	1·07	0·26	4·37	0·339	0·279,	0·399	61·1	58·4	1·05	0·40	2·74	0·368	0·267,	0·461
Protein (g)	59·5	58·1	1·02	0·32	3·26	0·344	0·283,	0·406	61·2	61·8	0·99	0·43	2·30	0·539	0·454,	0·614
Carbohydrate (g)	219·3	209·3	1·05	0·36	3·07	0·364	0·303,	0·425	221·5	219·3	1·01	0·52	1·96	0·575	0·495,	0·646
Total sugar (g)	97·7	86·1	1·14	0·17	7·39	0·398	0·339,	0·458	98·6	95·5	1·03	0·41	2·61	0·593	0·515,	0·662
NMES (g)	47·2	45·2	1·05	0·03	39·06	0·427	0·368,	0·487	57·2	52·0	1·10	0·16	7·70	0·627	0·553,	0·691
Alcohol (g)	0·01	0·02	0·58	0·00	1561	0·316	0·255,	0·379	0·03	0·02	1·29	0·00	4151	0·481	0·390,	0·563
Ca (mg)	733·5	701·3	1·05	0·28	3·88	0·427	0·367,	0·488	754·0	735·0	1·03	0·39	2·69	0·595	0·518,	0·663
Vitamin C (mg)	56·3	55·6	1·01	0·05	19·58	0·312	0·251,	0·374	75·4	68·4	1·10	0·18	6·70	0·478	0·386,	0·561
Fe (mg)	8·8	8·4	1·05	0·28	3·94	0·347	0·286,	0·409	9·0	9·1	1·00	0·42	2·38	0·516	0·428,	0·594

ICC, intra-class correlation coefficient; NMES, non-milk extrinsic sugars.

* Test–retest reliability of energy and nutrient intakes was assessed using data from three further UK-based studies where participants aged 11–88 years completed Intake24 a minimum of four times, as described in the Methods section.

† Reliability was estimated by linear mixed model.

Summaries of each nutrient for each age group and for all participants are given in Supplementary Table S2. The columns ‘lower’ and ‘upper’ refer to the lower and upper quartiles, respectively, of the corresponding nutrient.

Supplementary Table S3 shows how agreement varied by age group. The pairs of 24 h recalls gave values within 10 % of each other for energy and macronutrients, with the exception of alcohol where there was an 8 % difference for the 11- to 16-year-old group ranging up to a 76 % difference for the 16- to 24-year-old group.

ICC showed poor to moderate agreement in nutrient intakes. ICC for repeatability of a single recall are lower than for two recalls considered together, indicating large intra-individual variation. For example, for reported EI, ICC for a single recall was 0·347 and this increased to 0·516 when the repeatability of 2 d of recall was considered (Table 4). Alcohol is not included in the ICC analysis due to the large numbers of non-consumers; 86 % of the study population did not consume alcohol during the recording period.

Splitting the data by age group had little influence on the ICC. Supplementary Tables S4 and S5 report the ICC by age group, for a single recall and paired recalls, respectively, according to the model with sex as the only covariate, with 95 % CI, for each nutrient and each age group. ICC show poor to moderate agreement between single recalls for the majority of nutrients, with slight improvement when paired recalls are considered. Repeatability was best in the 65 years and over age group where ICC for intakes of NMES and Fe were moderate to excellent (0·863 and 0·857, respectively) and good to excellent (0·526 and 0·530, respectively), for paired recalls and single recalls, respectively.

## Discussion

In comparison with TEE measured in a cohort of UK adults aged 40–65 years, over 10–14 d using DLW, self-reported EI by Intake24 was underestimated by 25 % on average. The level of under-reporting was similar for men and women but was found to vary significantly with age, with older people tending to under-report to a lesser extent. Comparing the reported EI from a single recall with that from 2 or 3 d of recall, accuracy did not improve markedly with an increased number of days; however, the precision of estimates did improve, particularly with the second recall. Although accuracy of reporting improved with age, the intra-individual variation in under-estimation was constant across age groups. There was some evidence of systematic bias, with an increased tendency to under-report at lower levels of intake and to over-report at higher levels of intake, when reported EI from a single recall is considered, but this pattern disappeared when the mean of three recalls was used. This may be indicative of the day-to-day variation in EI and the need to collect data on multiple days.

Under-reporting of habitual EI may be due to under-reporting of food intakes during the recording period, under-eating during the recording period or a combination of the two. This has been examined using covert observation of individuals recording their food intake where a reduction in EI (under-eating) of 8 % in men and 3 % in women and under-reporting of 9 % by men and 12 % by women combined to give an under-estimate of around 15 % of EI^(26)^.

Average levels of under-reporting of total EI using Intake24 were similar to traditional dietary assessment methods implemented in surveys in the UK, the USA and elsewhere. The UK National Diet and Nutrition Survey Rolling Programme collects dietary data using a 4-d estimated weight food diary with interview. EI reported using this method was validated in a sub-sample of the population aged 4 years and over (*n* 371) against TEE assessed using DLW. EI was under-estimated on average across all age groups. The lowest levels of under-reporting were seen in the 4- to 10-year-old group where EI was under-estimated by 22 % on average. For participants aged 16 years and over mean under-estimates of EI ranged from 25 to 36 %^(27)^. Lopes *et al*.^(28)^ compared EI estimated by three interviewer-administered 24-h dietary recalls with TEE measured by DLW in eighty-three adults aged 20–60 years in Brazil. They found EI to be under-estimated by 23 % in men and by 40 % in women. A pooled analysis of five validation studies comparing 24-h dietary recalls with TEE measured by DLW found EI to be under-reported by 15 % on average, ranging from an under-estimate of 28 to 6 % for individual studies^(29)^. Few studies have reported the validity of EI assessed using online dietary systems against DLW-measured TEE. Reported EI based on six 24-h recalls completed using the web-based system DietDay was validated against DLW in 233 adults aged 21–69 years and EI was found to be under-reported by 10 % on average^(30)^. Comparison of a 4-d web-based food record with DLW-measured TEE in forty middle-aged adults in Sweden found that men under-reported EI by 24 % on average whereas women under-reported by 16 %^(31)^. EI reported using the online dietary recall system ASA24^®^^(5)^ was compared against DLW-measured TEE in older adults (mean age 62 years for women and 64 years for men)^(32)^. The average under-estimation of EI was 17 % in men (*n* 485) and 15 % in women (*n* 472), comparable with the 18·7 % under-estimation in our sample of 60-year-olds. Validation of EI against TEE using DLW assumes that participants are in energy balance. TEE is measured over a relatively short period, often 10–14 d and the weight change from a 500 kcal/d (2092 kJ/d) deficit over this period would only be around 500 g. Given the proportion of the population who are over-weight or obese, many participants may be making efforts to reduce their EI. Therefore, an EI:TEE ratio lower than 1·0 could represent accurate EI estimates to some extent.

In a study of 627 adults aged 50–70 years the reproducibility of a single dietary recall reported using ASA24^®^ was low, with ICC for energy and protein of 0·28 and 0·25, respectively^(33)^, slightly lower than the repeatability of a single recall using Intake24 (0·347 and 0·344). This difference may be due to the longer time between recalls in the ASA24^®^ study where recalls were repeated at 3-month intervals.

Assessing the reliability of measures of EI and nutrients via repeated 24-h recalls is complicated by the genuine day-to-day variation in individual food intakes. A way to address this is to collapse results of multiple recalls into pairs and test their reliability. Results of the Bland–Altman analysis showed good agreement between recalls 1 and 2 and recalls 3 and 4 for energy and macronutrients, but greater variability for alcohol and NMES. This may reflect greater day-to-day variability in intake of these nutrients, indicating that more than two recalls are required for accurate estimation of usual intakes for some nutrients^(34)^. Better reliability of intakes reported using Intake24 was observed in men and those aged 64 years and over, possibly suggesting less day-to-day variability in these individuals' diets.

### Study limitations and strengths

We have conducted a detailed analysis of misreporting of EI including how this varies by age, sex and BMI; however, the findings are not generalisable to people of ethnic minority groups, or from different socio-economic backgrounds, for whom the extent of misreporting may differ. Although the sample size for the DLW validation of Intake24 is relatively large for such studies, there was no consistency in the range of week and weekend days for which participants completed their recalls. The small number of days recalled is also a limitation but may reflect common choices in study designs. Energy expenditure was assessed over a 10–14 d period; for logistic reasons participants were free to choose the days to complete Intake24 and so may have avoided completing recalls on days they considered their intake to be unhealthy or too complicated to report, which would be likely to be days that EI was high.

TEE estimated using DLW requires a number of assumptions and inferences. These include that the individual is weight stable and that the levels of background isotope intake remain constant^(35,36)^. In this study, two pre-dose samples were taken which will reduce the associated error assigned to the variation in natural abundance^(36)^. Furthermore, as DLW only measures CO_2_ production and not directly O_2_ consumption, some knowledge of the energy equivalent of CO_2_ is needed for TEE estimation. This can be highly variable and macronutrient dependent. In the absence of a measurement of the respiratory quotient (RQ) which allows determining macronutrient oxidation, RQ was assumed to be 0·85, it being the average RQ of a standard Western diet. The RQ based on the dietary intake reported by our participants was 0·849 on average (sd 0·021) and given the known issues with under-reporting of food intakes rather than make any assumptions around the nutritional composition of ‘missing foods’ the fixed RQ was used. In total, the error associated with our calculations (2·04 ± 0·76 %) is well within the 2–8 % error deemed acceptable using the DLW method^(37)^.

Assessment of the repeatability of any short-term measure of dietary intake is complicated by the true day-to-day variation in individual intake. In our study we did not directly determine how much of the variation would be due to measurement error or true variability of food intakes. What the reliability results do indicate is the degree to which 2 d of recall is sufficient to obtain an estimate of habitual intake for a particular nutrient. At the population level, reported intakes of energy and macronutrients from one pair of non-consecutive 24 h recalls were within 10 % of those reported in a further pair of recalls completed by the same individual. At the individual level, however, there is much greater variation as evidenced by the wide limits of agreement and low ICC. However, week and weekend days were not balanced across the recall pairs, and therefore reliability estimates may be slightly attenuated for this reason. The repeatability analysis is conducted on pooled data from three studies that covered different age groups; while this is a strength in terms of generalisability of results, this also increases between-individual variation by which pooled ICC may be overestimated.

### Conclusions

Under-reporting of EI is a consistent finding when using dietary assessment methods which rely on self-reports of food and drink intake. We report that EI reported using Intake24 were under-estimated by around 25 % compared with TEE and were only weakly correlated.

From the reliability study, our findings indicate that 2 d of recalls using Intake24 are sufficient for the assessment of habitual intake of energy and macronutrients at the group level. More days are likely to be required for food components where day-to-day variation is greater, especially alcohol. The under-estimation of EI using Intake24 is comparable with more intensive methods of data collection such as interviewer-led 24 h recalls or estimated food diaries. As data are collected remotely, without the need for trained interviewers, and participants can complete recalls at a time convenient for them, the system offers a reduced cost and burden alternative for collecting dietary intake information. Future work should focus on whether the validity of self-reported methods such as Intake24 can be improved by combining these with image capture-based methods^(38–40)^ and/or mathematical modelling^(41,42)^.

## Supplementary material

For supplementary material accompanying this paper visit https://doi.org/10.1017/jns.2019.20. click here to view supplementary material
