# Validity and reliability of an online self-report 24-hour dietary recall method (Intake24): A doubly-labelled water study and repeated measures analysis — CORRIGENDUM

**DOI:** 10.1017/jns.2019.38

**Published:** 2019-12-19

**Authors:** Emma Foster, Clement Lee, Fumiaki Imamura, Stefanie E. Hollidge, Kate L. Westgate, Michelle C. Venables, Ivan Poliakov, Maisie K. Rowland, Timur Osadchiy, Jennifer C. Bradley, Emma L. Simpson, Ashley J. Adamson, Patrick Olivier, Nick Wareham, Nita G. Forouhi, Soren Brage

**Affiliations:** 1 Human Nutrition Research Centre, Institute of Health and Society, Newcastle University, Newcastle upon Tyne, UK; 2 School of Mathematics, Statistics and Physics, Newcastle University, Newcastle upon Tyne, UK; 3 Department of Mathematics and Statistics, Lancaster University, Lancaster, UK; 4 MRC Epidemiology Unit, University of Cambridge, Cambridge, UK; 5 MRC Elsie Widdowson Laboratory, Cambridge, UK; 6 Open Lab, School of Computing Science, Newcastle University, Newcastle upon Tyne, UK; 7 Faculty of Information Technology, Monash University, Clayton, VIC, Australia

**Keywords:** Dietary assessment, Online 24-h dietary recall, Doubly labelled water, Validation, Repeatability, Reliability, UK adults, Corrigendum

In the aforementioned article, the affiliation for Soren Brage is incorrect. The correct affiliation for Soren Brage is: MRC Epidemiology Unit, University of Cambridge, Cambridge, UK

The authors would like to apologise for this error.

## References

[ref1] Foster E., Lee C., Imamura F., (2019) Validity and reliability of an online self-report 24-h dietary recall method (Intake24): a doubly labelled water study and repeated-measures analysis. J Nutr Sci 8, E29. doi:10.1017/jns.2019.20 31501691 PMC6722486

