# Supplementary material for: Validity and reliability of an online self-report 24-h dietary recall method (Intake24): a doubly labelled water study and repeated-measures analysis
Source: J Nutr Sci. 2019 Aug 30;8:e29. doi: 10.1017/jns.2019.20 (PMC6722486; doi:10.1017/jns.2019.20)
Supplement: Supplementary file 1 [file S204867901900020Xsup001.docx]

**Supplementary Calculation S1**

*Total energy expenditure*

Rate constants and pool sizes are calculated from the slopes and intercepts of the log transformed data, with the rate of CO_2_ production, $R_{{CO}_{2}}$ given by([36](#_ENREF_36)):

$$R_{CO_{2}}=\frac{k_{O}N_{O}-k_{H}N_{H}-27.3\left( f_{2}-f_{1} \right)}{2f_{3}+2.1\left( f_{2}-f_{1} \right)}$$

Where k and N refer to the rate constant and pool size respectively, with subscripts to indicate the isotope. The fractionation factors f_1_, f_2_, and f_3_, are given as 0.941, 0.991 and 1.037 respectively.

$R_{{CO}_{2}}$ was converted to TEE using the energy equivalent of CO_2_ from Elia and Livesey([37](#_ENREF_37), [38](#_ENREF_38))with the Respiratory Quotient (RQ) fixed at 0.85 to reflect a typical Western diet:

$$TEE (kJ.{day}^{-1})=22.4\times\left( \frac{15.48}{RQ}+5.55 \right)R_{{CO}_{2}}(mol.{day}^{-1})$$

694 invitations sent

247 responses received

88 participants non-DLW

105 participants invited to do DLW*

100 participants completed DLW

- Did not pass pre-screening (n=13)
- Withdrew prior to booking (n=2)
- Unable to contact or attend appointment (n=36)

196 participants attended first clinic visit

- Did not pass screening (n=3)

193 participants

****Participants were allocated to DLW based on balancing sample across age, sex, BMI strata***

Excluded:

- Postponed appointment (n=3)
- Declined to do DLW (n=2)

93 participants enrolled into non-DLW arm of study (not reported in this paper)

Excluded from analysis (n=2):

Did not complete at least one Intake24 dietary recall during measurement period

**Included in DLW Validation study**

- Analysed datasets (n=98)
- Completed ≥ 2 24hr recalls (n=74)
- Completed ≥ 3 24hr recalls (n=53)

**Supplementary Fig. S1. Participant flow chart of the validation study**

**Study 3**

**Study 2**

**Study 1**

Previous participants of the Scottish Health Survey recruited by phone

Made contact

***n=*747**

Not eligible

***n=*71**

Agreed

***n=*384 (57%)**

Refused

***n=*239 (35%)**

Completed 4 or more recalls

***n=*133 (20%)**

Eligible

***n*=676 (100%)**

Unable

(due to illness/holiday)

***n=*53 (8%)**

Recruited via posters and leaflets displayed on campus, in shops and leisure centres.

Agreed

***n=*90**

Completed 4 or more recalls

***n=*46 (%)**

Completed 4 or more recalls

***n=*129 (44%)**

Agreed

***n=*291**

Recruited via letters home to secondary school children and on street recruitment.

**Included in Repeatability study**

Figure 1. Participant contact and response

**Supplementary Fig. S2. Participant flow chart for the reliability study**

Supplementary Fig. S3. Scatterplots of EI (kJ) against TEE (kJ) on original (left) and log (right) scale*

*The correlations presented do not truly reﬂect the relationship between EI and TEE because of intra-individual variation
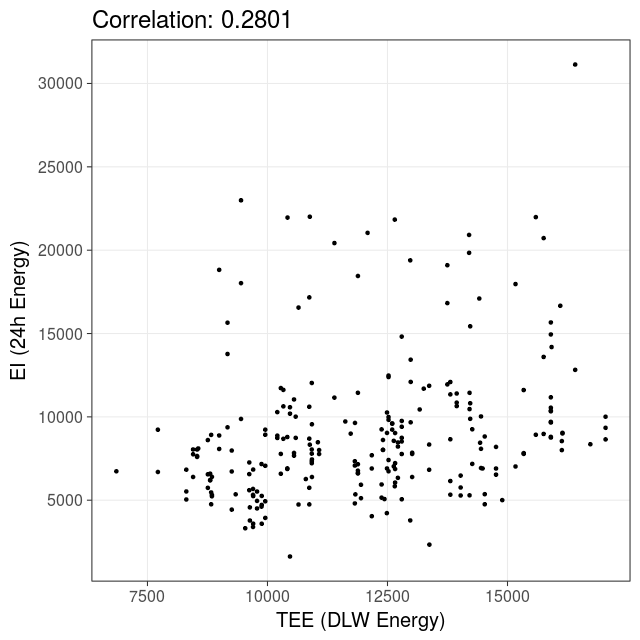

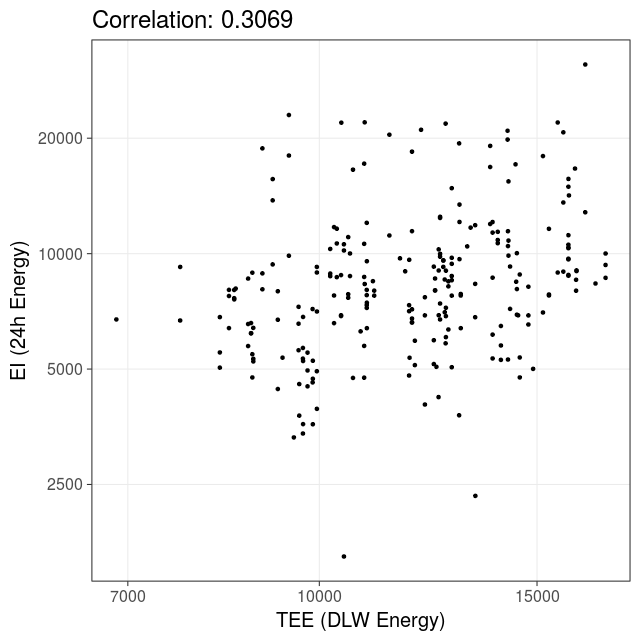
.

Supplementary Table S1. Estimates and credible intervals for applying the linear mixed effects models to different subsets of the data

|  | **Data** | **1 day** | **2 days (or fewer)** | **3 days (or fewer)** | **All** |
| --- | --- | --- | --- | --- | --- |
|  | Measurements | 98 | 172 | 225 | 247 |
| All covariates | (Intercept) | -0.1661  (-1.2377, 0.9139) | -0.3661  (-1.1522, 0.4367) | -0.1455  (-0.8073, 0.513) | -0.184  (-0.8091, 0.426) |
|  | Age | 0.0046  (-0.0007, 0.0099) | 0.0054  (0.0015, 0.0094) | 0.0063  (0.0031, 0.0096) | 0.0063  (0.0031, 0.0094) |
|  | Height (cm) | -0.0008  (-0.0067, 0.0052) | 0.0004  (-0.004, 0.0047) | -0.0009  (-0.0046, 0.0027) | -0.0009  (-0.0042, 0.0025) |
|  | BMI | -0.0035  (-0.015, 0.0078) | -0.0053  (-0.0139, 0.0036) | -0.0073  (-0.0147, 0.0001) | -0.006  (-0.013, 0.0011) |
|  | Gender (M) | -0.0354  (-0.1468, 0.0782) | -0.0302  (-0.114, 0.054) | -0.0013  (-0.0723, 0.0694) | -0.0026  (-0.0703, 0.0653) |
| Age only | (Intercept) | -0.4193  (-0.7137, -0.1266) | -0.4474  (-0.6593, -0.2387) | -0.4961  (-0.6762, -0.3168) | -0.4847  (-0.6552, -0.313) |
|  | Age | 0.0046  (-0.0007, 0.01) | 0.0055  (0.0016, 0.0093) | 0.0063  (0.0031, 0.0096) | 0.0061  (0.003, 0.0091) |

For each covariate, the standalone number represents the average effect on the validity measure. For example, for the ﬁrst day log-ratios, an increase in the age by 1 year corresponds to, on average, an increase of 0.0046 in the log-ratio. Whether such an effect is signiﬁcant is determined by whether 0 is in the 95% credible interval (CI), which is reported in the bracketed pair underneath.

Supplementary Table S2. Reliability analysis - Mean of 4 recalls, lower and upper quartiles of intake of energy and nutrients for all participants and split by age group

|  | **All ages** | | | **11-16 year olds** | | | **17-24 year olds** | | | **25-64 year olds** | | | **65 years +** | | |
| --- | --- | --- | --- | --- | --- | --- | --- | --- | --- | --- | --- | --- | --- | --- | --- |
|  | **Mean** | **Lower Upper** | | **Mean** | **Lower Upper** | | **Mean** | **Lower Upper** | | **Mean** | **Lower Upper** | | **Mean** | **Lower Upper** | |
| Energy (kJ) | 7600 | 5835 | 8849 | 7020 | 5497 | 8002 | 7985 | 6058 | 9416 | 7385 | 6087 | 8773 | 8329 | 6562 | 9146 |
| Fat (g) | 67.7 | 48.0 | 82.0 | 58.7 | 42.6 | 65.0 | 72.9 | 51.7 | 85.1 | 65.9 | 48.4 | 79.2 | 78.0 | 60.6 | 93.5 |
| Protein (g) | 66.9 | 50.3 | 78.5 | 56.6 | 44.1 | 67.2 | 70.1 | 52.9 | 80.3 | 71.3 | 56.7 | 79.8 | 75.5 | 62.9 | 94.2 |
| Carbohydrate (g) | 237.1 | 180.0 | 283.0 | 242.7 | 185.2 | 278.6 | 245.5 | 186.2 | 295.5 | 218.1 | 167.0 | 256.1 | 226.9 | 184.7 | 250.8 |
| Total Sugar (g) | 111.3 | 75.1 | 131.7 | 121.4 | 83.6 | 141.7 | 115.6 | 83.1 | 143.1 | 95.1 | 69.0 | 112.5 | 99.1 | 61.1 | 113.3 |
| NMES* (g) | 74.1 | 41.7 | 92.4 | 91.7 | 55.2 | 106.0 | 76.6 | 43.5 | 95.6 | 54.3 | 28.3 | 73.3 | 53.5 | 34.3 | 65.7 |
| Calcium (mg) | 829.8 | 590.9 | 997.4 | 792.7 | 551.6 | 953.7 | 818.7 | 593.8 | 992.6 | 871.8 | 649.5 | 1038.8 | 902.7 | 708.0 | 1099.3 |
| Vitamin C (mg) | 105.1 | 48.7 | 143.7 | 125.7 | 58.0 | 150.5 | 101.4 | 48.1 | 144.0 | 88.4 | 43.3 | 129.8 | 96.3 | 48.8 | 136.0 |
| Iron (mg) | 9.9 | 7.1 | 11.8 | 8.6 | 6.1 | 10.6 | 10.1 | 7.3 | 11.9 | 10.7 | 8.7 | 12.5 | 11.6 | 8.8 | 12.3 |

*NMES = non milk extrinsic sugar

Supplementary Table S3. Reliability analysis Bland-Altman analysis – Split by age group

| Age group | Nutrient | Recalls 1&2 Geometric mean | Recalls 3&4 Geometric mean | Mean  Ratio | Limits of Agreement  Lower Upper | |
| --- | --- | --- | --- | --- | --- | --- |
| 11-16 years | Energy (kJ) | 6686 | 6450 | 1.04 | 0.53 | 2.03 |
|  | Fat (g) | 54.6 | 51.4 | 1.06 | 0.42 | 2.65 |
|  | Protein (g) | 53.0 | 52.1 | 1.02 | 0.46 | 2.25 |
|  | Carbohydrate (g) | 228.9 | 221.5 | 1.03 | 0.50 | 2.13 |
|  | Total Sugar (g) | 109.3 | 102.3 | 1.07 | 0.43 | 2.66 |
|  | NMES* (g) | 76.8 | 70.5 | 1.09 | 0.28 | 4.28 |
|  | Alcohol (g) | 0.0 | 0.0 | 0.92 | 0.19 | 4.43 |
|  | Calcium (mg) | 745.8 | 664.2 | 1.12 | 0.43 | 2.95 |
|  | Vitamin C (mg) | 97.6 | 76.1 | 1.28 | 0.26 | 6.26 |
|  | Iron (mg) | 7.9 | 8.0 | 0.98 | 0.41 | 2.33 |
|  |  |  |  |  |  |  |
| 17-24 years | Energy (kJ) | 7741 | 7281 | 1.06 | 0.51 | 2.20 |
|  | Fat (g) | 66.9 | 60.9 | 1.10 | 0.39 | 3.07 |
|  | Protein (g) | 64.3 | 63.9 | 1.01 | 0.41 | 2.45 |
|  | Carbohydrate (g) | 233.0 | 228.7 | 1.02 | 0.50 | 2.07 |
|  | Total Sugar (g) | 106.3 | 100.6 | 1.06 | 0.42 | 2.67 |
|  | NMES* (g) | 65.4 | 57.7 | 1.25 | 0.09 | 17.45 |
|  | Alcohol (g) | 0.2 | 0.1 | 1.76 | 0.00 | >1000 |
|  | Calcium (mg) | 746.5 | 729.1 | 1.02 | 0.33 | 3.16 |
|  | Vitamin C (mg) | 69.4 | 65.8 | 1.05 | 0.15 | 7.34 |
|  | Iron (mg) | 9.3 | 9.2 | 1.00 | 0.39 | 2.60 |
|  |  |  |  |  |  |  |
| 25-64 years | Energy (kJ) | 6816 | 7034 | 0.97 | 0.54 | 1.75 |
|  | Fat (g) | 57.4 | 59.6 | 0.96 | 0.38 | 2.40 |
|  | Protein (g) | 63.4 | 68.3 | 0.93 | 0.41 | 2.12 |
|  | Carbohydrate (g) | 200.2 | 206.7 | 0.97 | 0.54 | 1.74 |
|  | Total Sugar (g) | 82.1 | 84.6 | 0.97 | 0.36 | 2.59 |
|  | NMES* (g) | 37.5 | 39.4 | 0.95 | 0.20 | 4.46 |
|  | Alcohol (g) | 3.1 | 3.4 | 1.42 | 0.00 | >1000 |
|  | Calcium (mg) | 742.4 | 800.4 | 0.93 | 0.44 | 1.96 |
|  | Vitamin C (mg) | 62.2 | 65.8 | 0.95 | 0.15 | 5.92 |
|  | Iron (mg) | 9.5 | 9.7 | 0.99 | 0.42 | 2.32 |
|  |  |  |  |  |  |  |
| 65 years + | Energy (kJ) | 8071 | 7714 | 1.05 | 0.59 | 1.85 |
|  | Fat (g) | 75.0 | 71.1 | 1.05 | 0.40 | 2.78 |
|  | Protein (g) | 72.7 | 70.2 | 1.04 | 0.45 | 2.41 |
|  | Carbohydrate (g) | 219.0 | 212.4 | 1.03 | 0.66 | 1.62 |
|  | Total Sugar (g) | 89.5 | 87.2 | 1.03 | 0.47 | 2.23 |
|  | NMES* (g) | 43.9 | 42.4 | 1.03 | 0.49 | 2.17 |
|  | Alcohol (g) | 24.1 | 18.4 | 0.74 | 0.00 | 712.6 |
|  | Calcium (mg) | 866.4 | 825.0 | 1.05 | 0.54 | 2.06 |
|  | Vitamin C (mg) | 83.0 | 62.7 | 1.32 | 0.25 | 6.99 |
|  | Iron (mg) | 10.8 | 10.3 | 1.05 | 0.62 | 1.79 |

Supplementary Table S4. Reliability analysis (ICC) of a single 24hr recall with sex as a covariate – by age group

|  | **11-16 year olds** | | | **17-24 year olds** | | | **25-64 year olds** | | | **65 years +** | | |
| --- | --- | --- | --- | --- | --- | --- | --- | --- | --- | --- | --- | --- |
|  | **ICC** | **95% CI** | | **ICC** | **95% CI** | | **ICC** | **95% CI** | | **ICC** | **95% CI** | |
| Energy (kJ) | 0.403 | 0.289 | 0.517 | 0.272 | 0.179 | 0.371 | 0.347 | 0.217 | 0.478 | 0.463 | 0.000 | 0.685 |
| Fat (g) | 0.334 | 0.220 | 0.452 | 0.337 | 0.242 | 0.436 | 0.312 | 0.183 | 0.446 | 0.001 | 0.000 | 0.003 |
| Protein (g) | 0.260 | 0.145 | 0.380 | 0.329 | 0.235 | 0.428 | 0.398 | 0.270 | 0.527 | 0.002 | 0.000 | 0.013 |
| Carbohydrate (g) | 0.417 | 0.306 | 0.532 | 0.301 | 0.207 | 0.400 | 0.338 | 0.207 | 0.473 | 0.449 | 0.231 | 0.659 |
| Total Sugar (g) | 0.498 | 0.388 | 0.605 | 0.370 | 0.276 | 0.466 | 0.187 | 0.013 | 0.329 | 0.525 | 0.319 | 0.717 |
| NMES* (g) | 0.490 | 0.381 | 0.597 | 0.435 | 0.343 | 0.528 | 0.113 | 0.001 | 0.270 | 0.526 | 0.318 | 0.716 |
| Calcium (mg) | 0.350 | 0.236 | 0.466 | 0.192 | 0.099 | 0.289 | 0.455 | 0.328 | 0.580 | 0.294 | 0.000 | 0.584 |
| Vitamin C (mg) | 0.504 | 0.398 | 0.609 | 0.349 | 0.254 | 0.448 | 0.323 | 0.194 | 0.457 | 0.286 | 0.001 | 0.550 |
| Iron (mg) | 0.338 | 0.224 | 0.455 | 0.182 | 0.088 | 0.279 | 0.350 | 0.221 | 0.483 | 0.530 | 0.321 | 0.722 |

*NMES = non milk extrinsic sugar

**Supplementary Table S5. Reliability analysis (ICC) of paired recalls (recalls 1&2 and recalls 3&4) by age group**

|  | **11-16 year olds** | | | **17-24 year olds** | | | **25-64 year olds** | | | **65 years +** | | |
| --- | --- | --- | --- | --- | --- | --- | --- | --- | --- | --- | --- | --- |
|  | **ICC** | **95% CI** | | **ICC** | **95% CI** | | **ICC** | **95% CI** | | **ICC** | **95% CI** | |
| Energy (kJ) | 0.591 | 0.436 | 0.712 | 0.370 | 0.200 | 0.518 | 0.631 | 0.480 | 0.746 | 0.697 | 0.420 | 0.856 |
| Fat (g) | 0.511 | 0.339 | 0.650 | 0.301 | 0.126 | 0.459 | 0.512 | 0.331 | 0.656 | 0.069 | -0.336 | 0.452 |
| Protein (g) | 0.427 | 0.239 | 0.584 | 0.465 | 0.306 | 0.599 | 0.691 | 0.559 | 0.790 | 0.169 | -0.258 | 0.534 |
| Carbohydrate (g) | 0.616 | 0.467 | 0.731 | 0.499 | 0.345 | 0.626 | 0.580 | 0.415 | 0.708 | 0.790 | 0.573 | 0.903 |
| Total Sugar (g) | 0.669 | 0.535 | 0.771 | 0.610 | 0.478 | 0.715 | 0.395 | 0.193 | 0.564 | 0.733 | 0.471 | 0.875 |
| NMES* (g) | 0.664 | 0.528 | 0.767 | 0.662 | 0.543 | 0.755 | 0.342 | 0.134 | 0.522 | 0.863 | 0.709 | 0.939 |
| Calcium (mg) | 0.462 | 0.281 | 0.611 | 0.231 | 0.047 | 0.400 | 0.717 | 0.592 | 0.808 | 0.682 | 0.391 | 0.849 |
| Vitamin C (mg) | 0.643 | 0.501 | 0.751 | 0.524 | 0.375 | 0.647 | 0.530 | 0.352 | 0.670 | 0.713 | 0.449 | 0.864 |
| Iron (mg) | 0.489 | 0.311 | 0.634 | 0.278 | 0.097 | 0.442 | 0.532 | 0.356 | 0.672 | 0.857 | 0.681 | 0.930 |

*NMES = non milk extrinsic sugar
